# Cold Rolling Texture Prediction Using Finite Element Simulation with Zooming Analysis

**DOI:** 10.3390/ma14226909

**Published:** 2021-11-16

**Authors:** Honghao Wang, Sheng Ding, Tom Taylor, Jun Yanagimoto

**Affiliations:** Department of Mechanical Engineering, The University of Tokyo, Hongo 7-3-1, Bunkyo, Tokyo 113-8656, Japan; wanghonghao.mech@gmail.com (H.W.); tom.taylor347@live.co.uk (T.T.); jun.52074.yanagimoto@cem.t.u-tokyo.ac.jp (J.Y.)

**Keywords:** cold rolling texture, finite element analysis, co-rotational formulation, Taylor model

## Abstract

Cold rolling is widely employed in the manufacturing industry for the production of metal plates. In the cold rolling process, the thickness reduction of the metal plate under the recrystallization temperature generates severe anisotropy; this influences the subsequent forming processes. Therefore, the generation and prediction of metal plate anisotropy during cold rolling is a highly interesting research topic involving upstream studies of sheet metal forming. In this study, using the finite element method with zooming analysis, we established an efficient elastic–plastic analysis method to predict the metal plate texture after cold rolling. This method for cold rolling texture prediction was confirmed by comparing the experimental and simulation results of cold rolling for an S45C plate with a body-centered cubic lattice. Further, the numerical analysis method proposed in this study can contribute to the study of anisotropy as an alternative to experimental approaches.

## 1. Introduction

Cold rolling is a widely used process in the manufacturing industry for metal plate production [1]. In this process, the thickness reduction of the metal plate under the recrystallization temperature generates severe anisotropy [2], which influences the sheet metal formation. Wang et al. [3] studied the tension leveling process using different constitutive models and observed that the plastic anisotropy influences the residual curvature of the leveled metal strip. Wu et al. [4] studied cup drawing and hole expansion processes using the Hill48 yield function under the associated and non-associated flow rules. They concluded that the plastic anisotropy has a significant influence on the sheet metal forming results, such as in cup height and thickness reductions. Friedman and Pan [5] predicted the forming limit curves for sheet metal forming using different yield criteria and concluded that the plastic anisotropy can influence the predicted results. Because the anisotropy generated from the cold rolling process significantly affects the subsequent forming processes [6], many researchers have studied the texture evolution of cold-rolled plates using numerical and experimental methods. Bate and Fonseca [7] predicted the texture development of steels during cold rolling using crystal plasticity models. Yanagimoto [8] proposed a transition probability model for active slip systems for body-centered cubic (BCC) metals to predict the texture evolution of steels after 50% cold rolling reduction. Additionally, Morimoto et al. [9] proposed a deformation texture prediction model comprising the Orowan and Taylor models to predict the rolling texture of steels. The texture predictions of the abovementioned studies were all compared with experimental results and presented acceptable prediction accuracy.

However, there is still room to improve the accuracy and efficiency of texture prediction. For instance, Das [10] adopted the Bayesian neural network to efficiently calculate the texture evolution of steels during cold rolling. Fujita et al. [11] adopted a hybrid crystal plasticity approach combining the finite element (FE) method and fast Fourier transform method to study the through-process texture evolution during plate rolling. Taking into account the development of intragranular misorientations, Després et al. [12] adopted the grain fragmentation visco-plastic self-consistent model to calculate the cold rolling texture of steels. The calculation accuracy was improved compared with the results when using the visco-plastic self-consistent model. Our study aimed to establish an efficient elastic–plastic analysis method to predict the metal plate texture after cold rolling using the FE method with zooming analysis. The FE method can calculate the stress and strain distributions of cold-rolled plates accurately, while the zooming analysis can calculate the cold rolling texture efficiently. The zooming analysis can be widely applied in various types of FE analysis to improve the calculation efficiency [13,14,15,16].

In this study, the stress and strain distributions of the cold-rolled plate were calculated at the macro scale first, then at the micro scale to simulate the cold-rolled texture using the previously obtained strain distribution as an input. In this way, the texture calculation was only performed in the magnified area, thereby improving the calculation efficiency. The predicted rolling texture can reflect the anisotropy of a cold-rolled plate and clarify the generation of anisotropy caused by the cold rolling process. Moreover, if the predicted rolling texture is sufficiently accurate, this method can be used in the study of metal plate anisotropy as an alternative to experimental methods such as electron backscatter diffraction (EBSD) and X-ray diffraction.

## 2. FE Modeling of Cold Rolling Process

In this section, the FE modeling of the rolls and cold-rolled plate, modeling of the normal and tangent contact behaviors between the rolls and cold-rolled plate, and co-rotational formulation for the FE analysis are introduced in detail.

### 2.1. Modeling of Rolls and Cold-Rolled Plate

**Figure 1:**
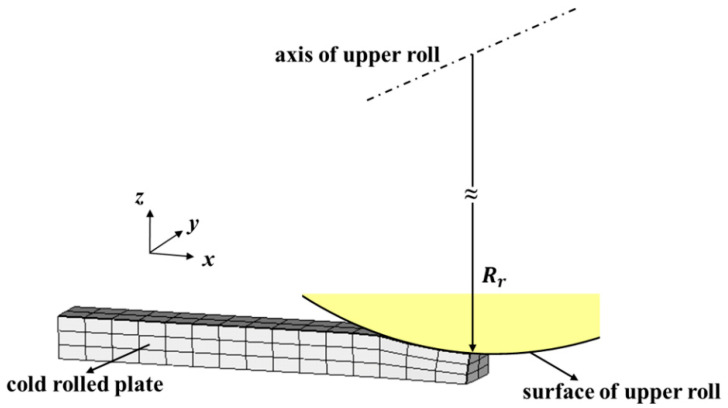
Schematic of the ¼ FE model for the cold rolling process.

For the FE modeling of the cold rolling process, the rigid analysis body was used to model the rolls and the first-order brick element with eight nodes (details in Appendix A) was adopted to model the cold-rolled plate. Figure 1 shows a schematic of the ¼ FE model for the cold rolling process considering the symmetry of the rolls and cold-rolled plate. In the model, x is the rolling direction (RD), y is the transverse direction (TD), and z is the thickness direction. The ½ surface of the upper roll is given by:

(1) $$SurRo\left(x,y,z\right)=\sqrt{{\left(x-x_{r}\right)}^{2}+{\left(z-z_{r}\right)}^{2}}=R_{r},\;\left(0\leq y\leq y_{r}\right)$$

where $R_{r}$ is the roll radius, $y_{r}$ is the half-width of the roll, and the line ($x=x_{r},z=z_{r})$ represents the upper roll axis.

**Figure 2:**
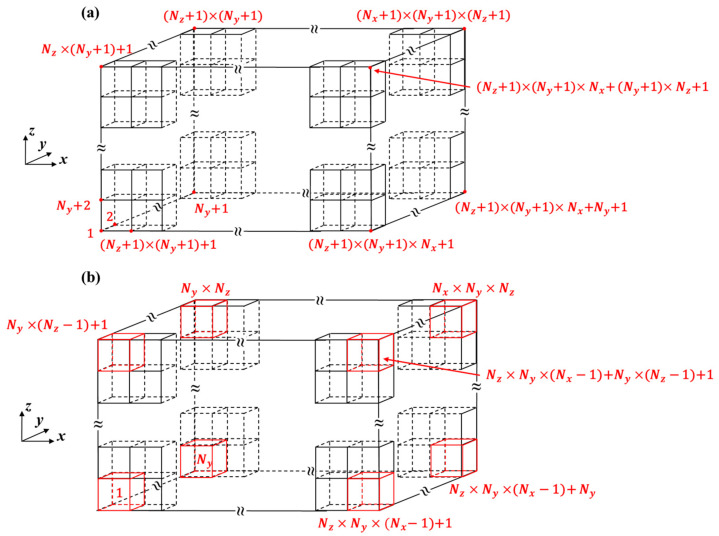
(**a**) Global node numbering and (**b**) element numbering schemes for cold-rolled plates.

Figure 2 shows the schematics of the global node numbering and element numbering for the cold-rolled plate, where $N_{x}$, $N_{y}$, and $N_{z}$ are the numbers of elements in the x, y, and z directions, respectively. Nodes and elements are numbered in the following order of direction: y→z→x. The global node number for node ($i_{nod}$, $j_{nod}$, $k_{nod}$) and the element number for the element ($i_{ele}$, $j_{ele}$, $k_{ele}$) are given by:

(2) $$\{\begin{array}{c} N_{Nod\left(i_{nod},\;j_{nod},\;k_{nod}\right)}=\left(i_{nod}-1\right)\left(N_{y}+1\right)\left(N_{z}+1\right)+\left(k_{nod}-1\right)\left(N_{y}+1\right)+j_{nod}\\ N_{Ele\left(i_{ele},\;j_{ele},\;k_{ele}\right)}=\left(i_{ele}-1\right)N_{y}N_{z}+\left(k_{ele}-1\right)N_{y}+j_{ele} \end{array}$$

where $i_{nod}$, $j_{nod}$, $k_{nod}$ and $i_{ele}$, $j_{ele}$, $k_{ele}$ represent the respective number positions of the node and element in the x, y, and z directions. To ensure an accurate FE analysis in the bite area, the mesh size in the rolling direction is determined by the thickness reduction of the cold-rolled plate and the length of the contact region between the roll and the plate. Additionally, the mesh sizes in the transverse and thickness directions were set to values in the same order of magnitude as the mesh size in the rolling direction to ensure calculation convergence.

### 2.2. Modeling of Contact

**Figure 3:**
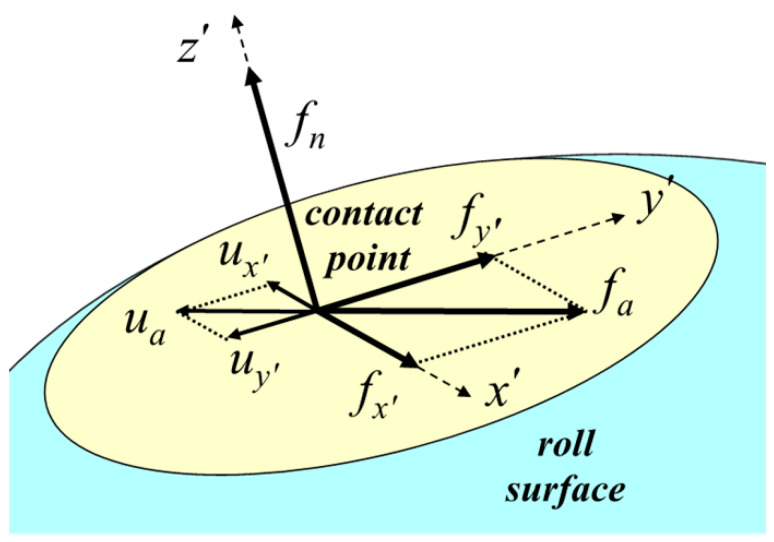
Schematic of the contact modeling.

For the contact modeling, the surface of the roll was treated as the master surface and the surface of the cold-rolled plate was treated as the slave surface. A schematic of the contact is shown in Figure 3, where $x^{\prime }$, $y^{\prime }$, and $z^{\prime }$ are the tangent, transverse, and normal directions to the roll surface of the contact point in the local coordinate system, respectively. The normal and tangent contact behaviors are calculated in the local coordinate system and then converted to the global coordinate system when solving the equilibrium equations of the FE analysis, which will be mentioned in Section 4 (details in Appendix B).

For normal contact behaviors, because the analytical rigid surface is adopted to model the roll surface as the master surface, the direct constraint elimination method can be applied by assuming a penetration of 0 to decide the location of the contact point of the cold-rolled plate in the $z^{\prime }$ direction. Subsequently, the rolling pressure $f_{n}$ can be calculated directly from the stress field of the contact element owing to the balance of internal and external forces in the normal direction [17].

For the tangent contact behaviors, we adopted the inverse tangent rule to calculate the friction between the roll and the cold-rolled plate in the $x^{\prime }$ and $y^{\prime }$ directions. The inverse tangent rule is given by:

(3) $$f_{a}=\left[\begin{array}{c} f_{x^{\prime }}\\ f_{y^{\prime }} \end{array}\right]=-\mu f_{n}\left(\frac{2}{\pi }\mathrm{artan}\left(\frac{u_{a}}{A}\right)\right)=\left[\begin{array}{c} -\mu f_{n}\left(\frac{2}{\pi }\mathrm{artan}\left(\frac{u_{x^{\prime }}}{A}\right)\right)\\ -\mu f_{n}\left(\frac{2}{\pi }\mathrm{artan}\left(\frac{u_{y^{\prime }}}{A}\right)\right) \end{array}\right]$$

where $f_{a}$ is the friction between the roll and cold-rolled plate, with components $f_{x^{\prime }}$ and $f_{y^{\prime }}$ in the $x^{\prime }$ and $y^{\prime }$ directions, respectively. Further, $u_{a}$ is the relative displacement of the contact point to the roll surface in the contact plane, with $u_{x’}$ and $u_{y’}$ being the respective relative displacements in the $x^{\prime }$ and $y^{\prime }$ directions, respectively. The factor $f_{n}$ is the rolling pressure normal to the roll surface, μ is the friction coefficient, and A is the parameter with an arbitrary value for numerical calculations. The recommended value range for the $\Delta u_{a}/A$ ratio is 100–1000 [18].

### 2.3. Co-Rotational Formulation

**Figure 4:**
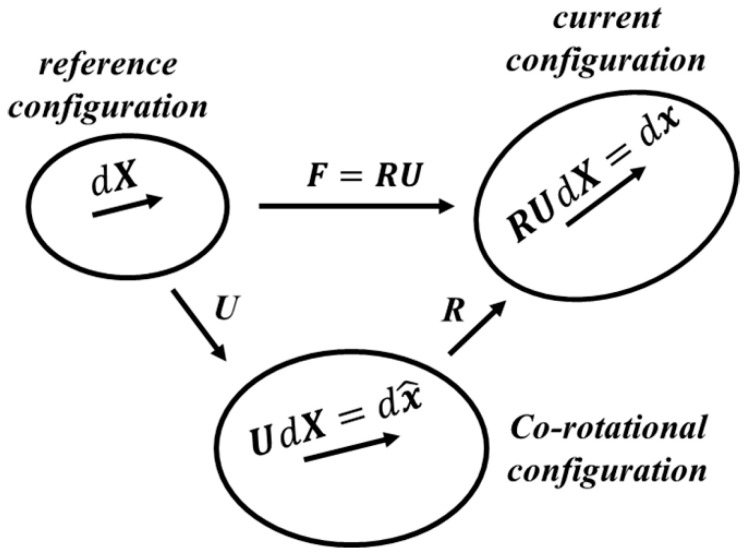
Schematic of the co-rotational formulation.

In this study, the co-rotational formulation was applied to define the strain in the elastic–plastic FE analysis of cold rolling to ensure the objectivity of the strain tensor [19]. Figure 4 shows the three configurations in the co-rotational formulation, which are defined according to the right polar decomposition of the deformation gradient tensor F, given by:

(4) F=RU

where R and U are the rotation and right stretch tensor, respectively. As shown in Figure 4, the configuration before deformation is set as the reference configuration and dX is the material differential fiber before deformation. Meanwhile, the current configuration is that after deformation, dX becomes dx=FdX considering the rotation and stretching in the current configuration. Thus, the deformation gradient tensor between the current configuration and reference configuration is defined as:

(5) $$F=\frac{dx}{dX}$$

The co-rotational configuration only considers the stretching, while the material differential fiber dX becomes $d\hat{x}=UdX$ in this configuration. As expressed in the following equations, the strain increment tensor $d\hat{\varepsilon }$ in the co-rotational configuration is defined by using the right stretch tensor U, while the strain increment tensor dε in the current configuration is given by considering the rigid body rotation.

(6) $$\{\begin{array}{cc} d\hat{\varepsilon }=\ln U\approx E-E^{2} & \mathrm{co}\text{-}\mathrm{rotational}\;\mathrm{configuration}\\ d\varepsilon =R\left(d\hat{\varepsilon }\right)R^{T}\approx R\left(E-E^{2}\right)R^{T} & \mathrm{current}\;\mathrm{configuration} \end{array}$$

The strain increment tensor $d\hat{\varepsilon }$ can then be calculated using the Green–Lagrange strain tensor E, while dε in the current configuration can be calculated using the Green–Lagrange strain tensor E and the rotation tensor R, where E and R are given as:

(7) $$\{\begin{array}{c} E=\left(F^{T}F-I\right)/2\\ R=FU^{-1}\approx F\left(I-E+3E^{2}/2\right) \end{array}$$

The elastic–plastic decomposition of the strain increment tensor in the co-rotational formulation is given by:

(8) $$\{\begin{array}{cc} d\hat{\varepsilon }=d{\hat{\varepsilon }}^{e}+d{\hat{\varepsilon }}^{p} & \mathrm{co}\text{-}\mathrm{rotational}\;\mathrm{configuration}\\ d\varepsilon =d\varepsilon^{e}+d\varepsilon^{p} & \mathrm{current}\;\mathrm{configuration} \end{array}$$

where $d{\hat{\varepsilon }}^{e}$ and $d{\hat{\varepsilon }}^{p}$ are the elastic and plastic strain increment tensors observed in the co-rotational configuration, respectively. Meanwhile, the corresponding strain increment tensors observed in the current configuration are denoted without carets. In the next section, material constitutive modeling at both the macro and micro scales is introduced under the co-rotational and current configurations of the co-rotational formulation.

## 3. Macro-Scale and Micro-Scale Material Constitutive Modeling

**Figure 5:**
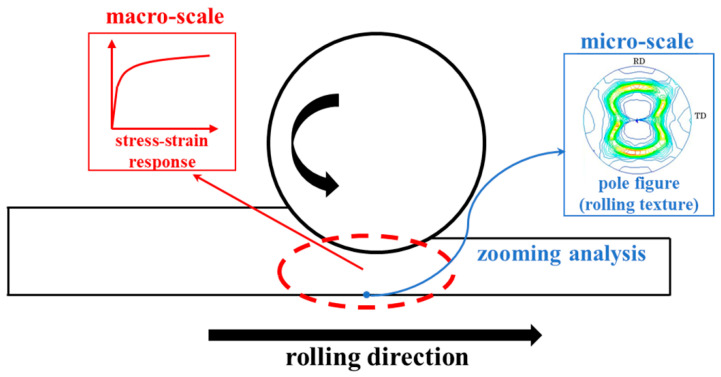
Schematic of the current analysis framework.

To predict the cold rolling texture of the metal plate, a macro-scale phenomenological constitutive model and a micro-scale crystal plasticity constitutive model were incorporated into the current elastic–plastic analysis framework, as shown in Figure 5. The macro-scale model was applied to the elastic–plastic FE analysis of cold rolling to predict the stress and strain distributions of the cold-rolled plates, while the micro-scale model was coupled with the macro-scale model to predict the local cold rolling texture using zooming analysis. The details of the current analysis framework are described in this section.

### 3.1. Macro-Scale Phenomenological Constitutive Model for Stress and Strain Calculation

We defined the macro-scale phenomenological constitutive model of a cold-rolled plate in the co-rotational configuration. For the elastic domain, this model is given by:

(9) $$d\hat{\sigma }=\left[\begin{array}{cccccc} \lambda +2G & \lambda & \lambda & 0 & 0 & 0\\ \lambda & \lambda +2G & \lambda & 0 & 0 & 0\\ \lambda & \lambda & \lambda +2G & 0 & 0 & 0\\ 0 & 0 & 0 & G & 0 & 0\\ 0 & 0 & 0 & 0 & G & 0\\ 0 & 0 & 0 & 0 & 0 & G \end{array}\right]d{\hat{\varepsilon }}^{e}$$

where λ=(Eν)/[(1−2ν)(1+ν)] is the Lamé constant and G=E/[2(1+v)] is the shear modulus of elasticity. In both terms, E is the Young’s modulus and ν is Poisson’s ratio.

For the plastic domain, the macro-scale phenomenological constitutive model comprises the von Mises yield criterion (Equation (10)), associated flow rule (Equation (11)), and Kim–Tuan isotropic hardening rule (Equation (12)) for an accurate description of the flow stress for large deformation [20]. The reason for adopting these three constitutive relations is because cold rolling is a relatively simple manufacturing process compared with some sheet metal forming processes; that is, for the work-hardening rule, there is no loading reversal during the cold rolling process. Therefore, in this study, it is not necessary to adopt some of the more complicated work-hardening rules (i.e., there is no need to consider the effect of material properties, such as the Bauschinger effect [21]). The isotropic hardening rule together with the adopted yield criterion and flow rule are sufficient to describe the macro-scale material responses during cold rolling.

(10) $$f\left(\hat{\sigma }\right)=\sqrt{\frac{{\left({\hat{\sigma }}_{yy}-{\hat{\sigma }}_{zz}\right)}^{2}+{\left({\hat{\sigma }}_{zz}-{\hat{\sigma }}_{xx}\right)}^{2}+{\left({\hat{\sigma }}_{xx}-{\hat{\sigma }}_{yy}\right)}^{2}+6{\hat{\sigma }}_{yz}^{2}+6{\hat{\sigma }}_{zx}^{2}+6{\hat{\sigma }}_{xy}^{2}}{2}}$$

(11) $$\;d{\hat{\varepsilon }}^{p}=\frac{\partial f\left(\hat{\sigma }\right)}{\partial \hat{\sigma }}d\lambda$$

(12) $$\bar{\sigma }=f\left(\hat{\sigma }\right)=\sigma_{y}+K(1-e^{-t{\bar{\varepsilon }}^{p^{a}}}){\bar{\varepsilon }}^{p^{h}}$$

where $\hat{\sigma }$ is the Cauchy stress tensor in the co-rotational configuration, dλ is the plastic multiplier, and ${\bar{\varepsilon }}^{p}$ is the equivalent plastic strain. The parameters $\sigma_{y}$, K, t, a, and h of the Kim-Tuan isotropic hardening rule can be identified from the strain-stress curve obtained from the compression test in the thickness direction. It should be noted that this macro-scale constitutive model is defined in the co-rotational configuration of the formulation mentioned in Section 2.3; thus, to obtain the stress and strain distributions in the current configuration, the rigid body rotation must be considered. The macro-scale constitutive model is applied to the FE analysis of cold rolling to predict the stress and strain distributions for the cold-rolled plate (discussed in Section 4).

### 3.2. Micro-Scale Crystal Plasticity Constitutive Model for Lattice Orientation Calculation

The Taylor model [22] was adopted as the micro-scale crystal plasticity constitutive model to predict the cold rolling texture in the current configuration of the co-rotational formulation. This model is widely applied to calculate the texture evolution during metal forming processes for BCC [9], face-centered cubic [23], and hexagonal close-packed [24] metals owing to its simplicity and accuracy. To predict the texture evolution of the cold-rolled plate using zooming analysis, the Taylor model, coupled with the macro-scale phenomenological constitutive model mentioned in Section 3.1, is given by:

(13) $$\tau^{\left(k\right)}=\sigma :P^{\left(k\right)}$$

(14) $$d\varepsilon^{p}=\underset{k}{\sum }P^{\left(k\right)}d\gamma^{\left(k\right)}$$

(15) $$P^{\left(k\right)}=\frac{1}{2}\left(a_{i}^{\left(k\right)}b_{j}^{\left(k\right)}+a_{j}^{\left(k\right)}b_{i}^{\left(k\right)}\right)e_{i}⊗e_{j}$$

where $\tau^{\left(k\right)}$ is the shear stress of the slip system *k*, $d\varepsilon^{p}$ is the plastic strain increment tensor, and σ is the Cauchy stress tensor in the current configuration of the co-rotational formulation. Moreover, $P^{\left(k\right)}$ is the symmetric part of the Schmid tensor of the slip system *k*, $d\gamma^{\left(k\right)}$ is the slip increment of the slip system, $e_{i}$ is the orthonormal basis vector, $a^{\left(k\right)}$ is the unit normal vector of the slip plane, and $b^{\left(k\right)}$ is the unit slip direction vector. The yield condition for the slip system is given by:

(16) $$\max\left(\frac{\left|\tau^{\left(k\right)}\right|}{\tau_{s}{}^{\left(k\right)}}\right)=1$$

where $\tau_{s}{}^{\left(k\right)}$ is the critical resolved shear stress (CRSS) of the slip system. When $\left|\tau^{\left(k\right)}\right|=\tau_{s}{}^{\left(k\right)}$, the slip of slip system begins. Considering Equations (13)–(15), the grain plastic work increment is given by:

(17) $$dW^{p}=\underset{k}{\sum }\tau_{s}{}^{\left(k\right)}d\gamma^{\left(k\right)}$$

Based on the minimum slip principle [22], the grain plastic work increment $dW^{p}$ is minimized using the interior point legacy algorithm [25] under the constraints of Equations (14) and (15) to determine the slip for all slip systems. The lattice orientations can then be calculated using the slip values $d\gamma^{\left(k\right)}$ for all slip systems to predict the local cold rolling texture via zooming analysis (details in Section 4).

## 4. FE and Zooming Analysis Method for Cold Rolling Texture Prediction

In this study, the cold rolling process was analyzed as a static problem; thus, the static implicit method was applied to the FE analysis of cold rolling using the co-rotational formulation mentioned in Section 2.3. The equilibrium equation, based on the principle of virtual work using the co-rotational formulation, is given by:

(18) $$\{\begin{array}{c} \int_{V}\Pi :\delta FdV=\int_{S_{t}}t\delta udS\\ \Pi =\left(\det F\right)U^{-1}\hat{\sigma }R^{T}\\ \delta F=\frac{\partial \delta u}{\partial X} \end{array}$$

where Π is the first Piola–Kirchhoff stress tensor, *V* is the volume of the cold-rolled plate, t is the surface traction imposed on the cold-rolled plate, δu is the virtual displacement vector, and $S_{t}$ is the surface region where the surface traction is imposed. Here, X represents the coordinates of the reference configuration. Using the FE discretization for the cold-rolled plate mentioned in Section 2.1, the displacement vector u in an element is

(19) $$u=Nu^{\left(nod\right)}$$

where N is the shape function of the element (details in Appendix A) and $u^{\left(nod\right)}$ is the nodal displacement vector of a node in the element. Thus, the FE form of the equilibrium equation for an element is given by:

(20) $$\int_{V^{e}}\Pi \frac{\partial N}{\partial X}\delta u^{\left(nod\right)}dV=\int_{S_{t}^{e}}tN\delta u^{\left(nod\right)}dS$$

where $V^{e}$ and $S_{t}^{e}$ represent the volume and surface subjected to the surface traction of the element, respectively. Dropping the nodal virtual displacement $\delta u^{\left(nod\right)}$ on both sides of Equation (20) gives:

(21) $$\int_{V^{e}}\Pi \frac{\partial N}{\partial X}dV=\int_{S_{t}^{e}}tNdS\equiv {\left(T^{\left(nod\right)}\right)}_{S_{t}^{e}}$$

where $T^{\left(nod\right)}$ is the equivalent nodal force imposed on the node. In the FE analysis of cold rolling, $T^{\left(nod\right)}$ comprises the rolling pressure and friction mentioned in Section 2.2. Since Equation (21) is a nonlinear equation for the displacement vector u, the Newton–Raphson method was applied to solve for the u as an implicit method. By applying the Newton–Raphson method, we obtained the linearized form of Equation (21), which is called the element tangent stiffness equation, given by:

(22) $$\left[k^{e}\right]\Delta u=\left\{t^{e}\right\}-\left\{q^{e}\right\}$$

where:

(23) $$\{\begin{array}{c} \left[k^{e}\right]=\left(\int_{V^{e}}\frac{\partial \Pi }{\partial u}\frac{\partial N}{\partial X}dV-{\left(\frac{\partial T^{\left(nod\right)}}{\partial u}\right)}_{S_{t}^{e}}\right)\\ \left\{t^{e}\right\}={\left(T^{\left(nod\right)}\right)}_{S_{t}^{e}}\\ \left\{q^{e}\right\}=\int_{V^{e}}\Pi \frac{\partial N}{\partial X}dV \end{array}$$

The element tangent stiffness equation is used to assemble the corresponding global tangent stiffness equations [K]Δu={T}−{Q} according to the global node and element numbering given by Equations (19) and (20). The equation is then used to solve Δu for all nodes in the $m^{th}$ iteration of the Newton–Raphson method (Equation (24)).

(24) $${\left[K\right]}^{\left(m-1\right)}\Delta u^{\left(m\right)}=\left\{T^{\left(m-1\right)}\right\}-\left\{Q^{\left(m-1\right)}\right\}$$

The global tangent stiffness equations are stored using the skyline method and the modified Cholesky method is adopted to solve the equations because of its compatibility with the skyline method [26]. When solving Equation (21) using the Newton–Raphson method, after obtaining $\Delta u^{\left(m\right)}$ in the $m^{th}$ iteration by solving Equation (24), the displacement $u^{\left(m\right)}$ in the $m^{th}$ iteration is given by:

(25) $$u^{\left(m\right)}=u^{\left(m-1\right)}+\beta \Delta u^{\left(m\right)}$$

where β is a relaxation coefficient that promotes convergence of the calculation. For the convergence judgment of the Newton–Raphson method, the following residuals were used:

(26) $$\{\begin{array}{c} R_{1}=\frac{\left\|\Delta u^{\left(m\right)}\right\|}{\left\|u^{\left(m\right)}\right\|}<Tol1\\ R_{2}=\left\|\{T^{\left(m-1\right)}\right\}-\left\{Q^{\left(m-1\right)}\}\right\|<Tol2 \end{array}$$

**Figure 6:**
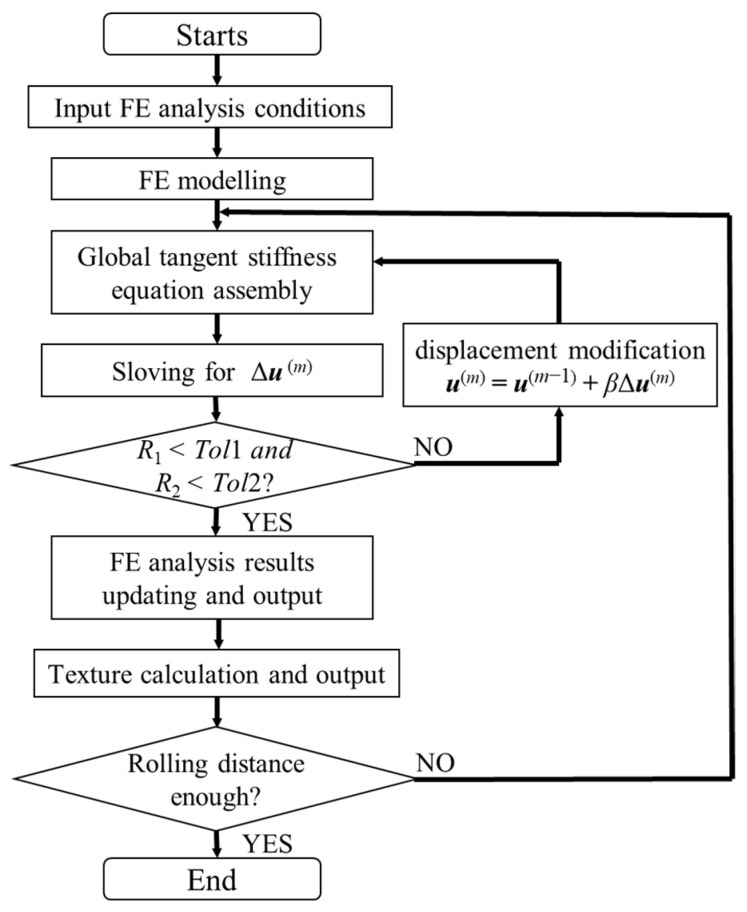
Flowchart of FE analysis for cold rolling texture prediction using zooming analysis.

Figure 6 shows a flowchart of the FE analysis for cold rolling texture prediction using zooming analysis. For the calculation of the stress and strain distributions during the assembly of the global tangent stiffness equation, we adopt the return mapping algorithm as the stress integration scheme to calculate the stress and strain in the co-rotational configuration using the macro-scale constitutive model mentioned in Section 3.1. For each element, the strain is calculated using the selective reduced integration with eight Gaussian integration points and an integration point located at the element center to avoid volumetric locking [27]. For the calculation of the cold rolling texture, the local plastic strain increment obtained by the FE analysis, which is represented by the nodal plastic strain increment in the corresponding area, is selected to be substituted into Equation (14) for the zooming analysis. The texture calculation, as shown in Figure 6, can then be conducted with the micro-scale constitutive model mentioned in Section 3.2 using the nodal plastic strain increment as an input. The selection of the slip systems and the corresponding CRSS ratios for the texture calculation depends on the cold rolled materials, which is discussed in detail in Section 5.

## 5. Validation of Current Analysis Framework

To validate the proposed elastic–plastic analysis framework under the co-rotational formulation, we conducted a cold rolling experiment with a 28.33% thickness reduction for an S45C plate (Nippon Steel, Tokyo, Japan) with an 84 mm width and 6 mm thickness. The FE analysis and texture prediction for the cold rolling process were then conducted for comparison using the analysis method mentioned in Section 4.

**Figure 7:**
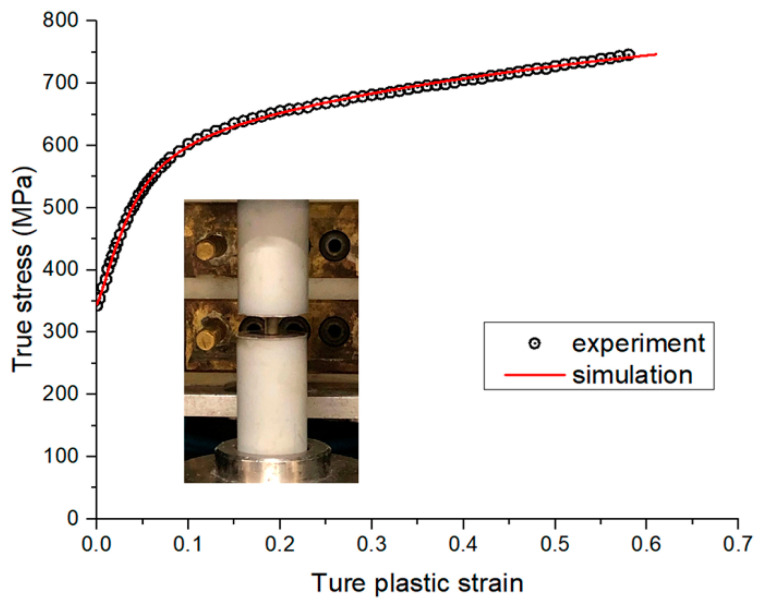
Experimental and simulation flow stress of the S45C plate obtained using the compression test.

The FE modeling parameters were matched with the experimental cold rolling conditions, while the detailed FE analysis conditions for the S45C plate are listed in Table 1. The ¼ plate was discretized using 3840 elements (i.e., 160, 8, and 3 grids along the rolling, width, and thickness directions were generated, respectively). It is worth mentioning that a case study was conducted to finally determine the appropriate mesh size under the premise of guaranteeing both the calculation efficiency and accuracy. Before cold rolling, the flow stress curve of the S45C plate was obtained first by conducting compression tests in the thickness direction using cylindrical S45C plate samples with a diameter of 4 mm and a height of 6 mm. Two compression tests were conducted and the obtained flow stress curves were identical (experimental results are shown in Figure 7). For the elastic deformation, the Young’s modulus and Poisson’s ratio were set to 210 GPa and 0.3, respectively. For the plastic deformation, the Kim–Tuan isotropic work-hardening rule (Equation (12)) was adopted to fit the experimental flow stress curve, and the results are shown in Figure 7. Moreover, the friction coefficient of the inverse tangent rule (Equation (3)) is assumed to be 0.3.

**Figure 8:**
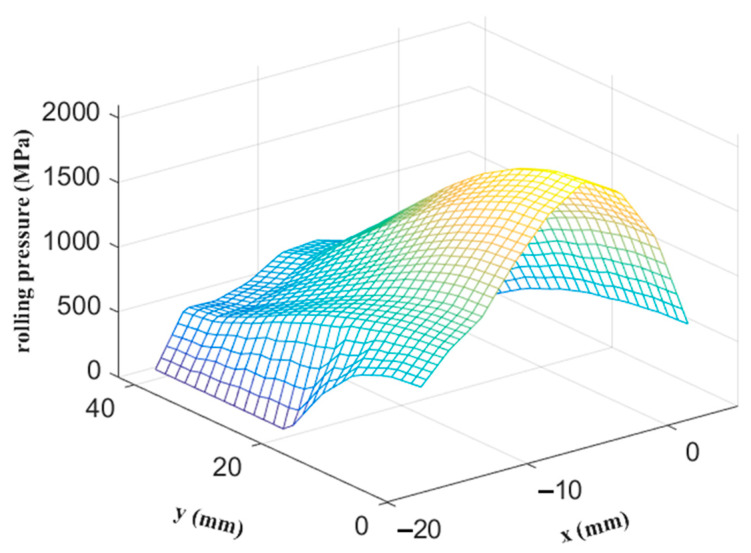
Distribution of rolling pressure obtained using FE analysis in the contact area.

**Figure 9:**
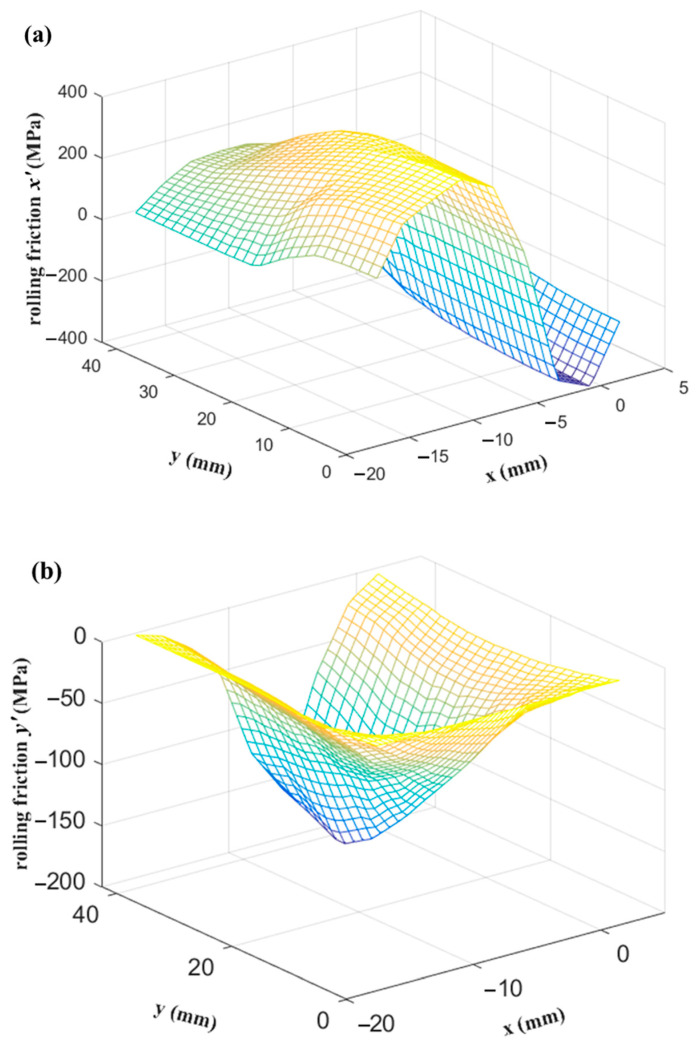
Distribution of rolling friction obtained using FE analysis in the contact area in the (**a**) $x^{\prime }$ direction and (**b**) $y^{\prime }$ direction.

The distributions of the rolling pressure and rolling friction in the contact area calculated by the FE analysis are shown in Figure 8 and Figure 9, respectively. The neutral line of the friction in the $x^{\prime }$ direction was successfully predicted by the inverse tangent rule. Moreover, the position of the peak rolling pressure is near the neutral line, which is consistent with the results obtained by Richelsen and Tvergaard [28]. The experimental and simulation results of the width spread ratio, rolling force, and rolling torque are shown in Table 2, highlighting the accuracy of the proposed elastic–plastic FE analysis method.

For the texture prediction, because of the BCC lattice structure of the cold-rolled S45C plate, 48 slip systems (listed in Table 3) were considered for the Taylor model to calculate the cold rolling texture. Because the {110}<111> and {112}<111> slip systems contribute equally to the deformation and the {123}<111> slip systems are not active during the cold forming process, the CRSS ratios among the {110}<111>, {112}<111>, and {123}<111> slip systems were set to 1:1:10 [29].

A node at the central layer of the center part of the cold-rolled S45C plate was selected for the validation of the texture prediction. The texture of the corresponding area was obtained by EBSD measurements for comparison with the prediction results. At the central layer of the cold-rolled plate, the lattice rotation tensor $d\omega^{\left(k\right)}$ caused by the slip of slip system *k* is given by:

(27) $$d\omega^{\left(k\right)}=-d\gamma^{\left(k\right)}Q^{\left(k\right)}/2$$

where $Q^{\left(k\right)}$ is the unsymmetric part of the Schmid tensor of slip system *k* and is expressed as:

(28) $$Q^{\left(k\right)}=\left(a_{i}^{\left(k\right)}b_{j}^{\left(k\right)}-a_{j}^{\left(k\right)}b_{i}^{\left(k\right)}\right)e_{i}⊗e_{j}$$

The total lattice rotation tensor of the calculated node in the central layer is the sum of the lattice rotations of all slip systems. Considering all the lattices contained by the calculated node, the lattice orientations can be calculated using Equations (27) and (28) to predict the cold rolling texture.

**Figure 10:**
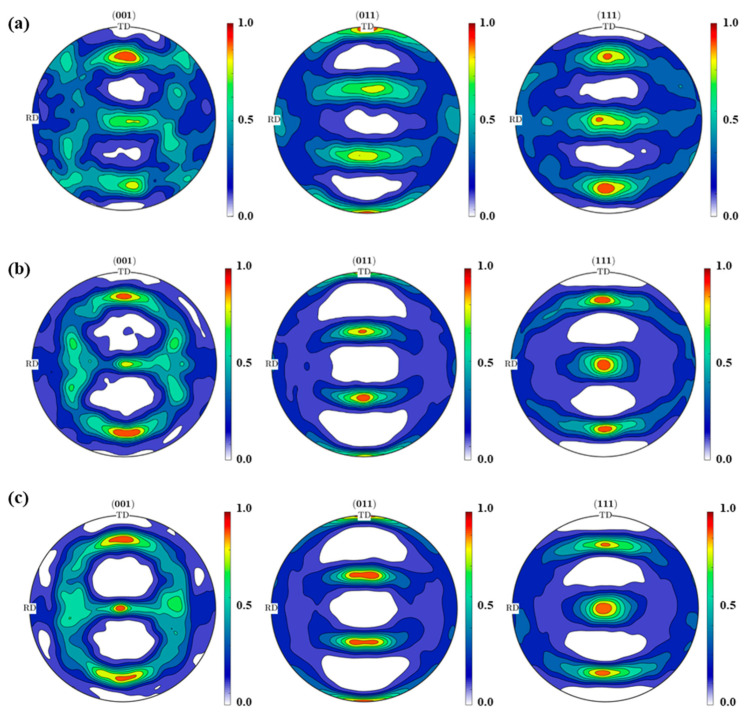
Cold rolling texture at the central layer of the center part of the S45C plate obtained by (**a**) EBSD measurement, (**b**) simulation with a CRSS ratio of 1:1:10, and (**c**) simulation with a CRSS ratio of 1:1:1.

Figure 10a shows the EBSD results. The measurements obtained 4260 lattice orientations in a scan area of 2 mm^2^ in the central layer of the center part of the cold-rolled plate. Comparing this with the texture prediction, we calculated the same number of random original lattice orientations using the Taylor model and the nodal plastic strain increment obtained using FE analysis. The predicted texture is shown in Figure 10b, and the simulation result with a CRSS ratio of {110}<111>:{112}<111>:{123}<111> = 1:1:1 is also shown in Figure 10c for comparison. The proposed elastic–plastic analysis framework predicts well the (001), (011), and (111) pole figures with normalized density. The simulation with a CRSS ratio of {110}<111>:{112}<111>:{123}<111> = 1:1:10 provides a slightly higher accuracy for the (001) pole figure in the RD than the simulation with a CRSS ratio of {110}<111>:{112}<111>:{123}<111> = 1:1:1, indicating that it is reasonable to set a relatively higher CRSS for the {123}<111> slip system during the cold rolling of the S45C plate. This was also validated by Yanagimoto [8] using the transition probability model for active slip systems for BCC metals. It is worth mentioning that the pole figure with normalized density can be used for the prediction of directional R-values [30] and yield stresses [31], which are important manifestations of anisotropy; thus, the proposed analysis framework can contribute to the studies of the anisotropy generation and prediction related to the cold rolling process. It is also a potential alternative to experimental measurements of cold rolling texture.

## 6. Conclusions

We proposed an elastic–plastic analysis framework, which incorporated a macro-scale FE analysis, using a phenomenological constitutive model for stress and strain calculations and a micro-scale zooming analysis using a crystal plasticity constitutive model for lattice orientation calculation. The proposed framework was applied to the elastic–plastic FE analysis of cold rolling using the static implicit method under a co-rotational formulation to predict the local cold rolling texture.

The cold rolling texture between the predicted results and EBSD measurements on the S45C plate was in good agreement, thereby validating the proposed elastic–plastic analysis framework. Therefore, the proposed method qualifies as an effective numerical method for future studies on anisotropy generation and prediction related to the cold rolling process. It is also a potential alternative to experimental measurements of cold rolling texture. Further improvement of the current analysis framework may include adding feedback from the micro scale to the macro scale (e.g., adopting an anisotropic constitutive model at the macro scale and calculating the anisotropic parameters using the predicted texture at the micro scale as the feedback) for multi-scale analysis, which will be the focus of our future studies.

## Figures and Tables

**Table 1:** FE analysis conditions for the S45C plate.

Material	S45C
Roll radius	180 mm
Plate dimensions	320 mm (length) × 84 mm (width) × 6 mm (thickness)
Plate mesh size	2.00 mm (length) × 5.25 mm (width) × 1.00 mm (thickness)
Thickness reduction	28.33%
Young’s modulus	210 GPa
Poisson’s ratio	0.3
Flow stress	$\bar{\sigma }=344.98+452.09\left(1-e^{-42.01{\bar{\varepsilon }}^{p^{1.04}}}\right){\bar{\varepsilon }}^{p^{0.24}}$ MPa
Friction coefficient	0.3

**Table 2:** Comparison of experimental and simulation results for the S45C plate.

	Width Spread Ratio	Rolling Force	Rolling Torque
Experiment	1.19%	2058 kN	17.7 kN·m
Simulation	1.19%	1870 kN	13.8 kN·m

**Table 3:** Slip systems for the S45C plate.

{110}<111>	{112}<111>	{123}<111>	
( 1 1 0)[−1 1 1]	( 1 1 2)[−1−1 1]	( 1 2 3)[ 1 1−1]	( 2 3 1)[ 1−1 1]
( 1 1 0)[ 1−1 1]	(−1−1 2)[ 1 1 1]	(−1 2 3)[ 1−1 1]	(−2 3 1)[ 1 1−1]
(−1 1 0)[ 1 1−1]	(−1 1 2)[ 1−1 1]	( 1−2 3)[−1 1 1]	( 2−3 1)[ 1 1 1]
(−1 1 0)[ 1 1 1]	( 1−1 2)[−1 1 1]	( 1 2−3)[ 1 1 1]	( 2 3−1)[−1 1 1]
( 1 0 1)[ 1 1−1]	( 1 2 1)[−1 1−1]	( 2 1 3)[ 1 1−1]	( 3 2 1)[−1 1 1]
( 1 0 1)[−1 1 1]	(−1 2−1)[ 1 1 1]	(−2 1 3)[ 1−1 1]	(−3 2 1)[ 1 1 1]
(−1 0 1)[ 1−1 1]	( 1 2−1)[ 1 1 1]	( 2−1 3)[−1 1 1]	( 3−2 1)[ 1 1−1]
(−1 0 1)[ 1 1 1]	(−1 1 2)[ 1 1−1]	( 2 1−3)[ 1 1 1]	( 3 2−1)[ 1−1 1]
( 0 1 1)[ 1 1−1]	( 2 1 1)[ 1−1−1]	( 1 3 2)[ 1−1 1]	( 3 1 2)[−1 1 1]
( 0 1 1)[ 1−1 1]	( 2−1−1)[ 1 1 1]	(−1 3 2)[ 1 1−1]	(−3 1 2)[ 1 1 1]
( 0−1 1)[ 1 1 1]	( 2−1 1)[ 1 1−1]	( 1−3 2)[ 1 1 1]	( 3−1 2)[ 1 1−1]
( 0−1 1)[−1 1 1]	( 2 1−1)[ 1−1 1]	( 1 3−2)[−1 1 1]	( 3 1−2)[ 1−1 1]

**Figure A1:**
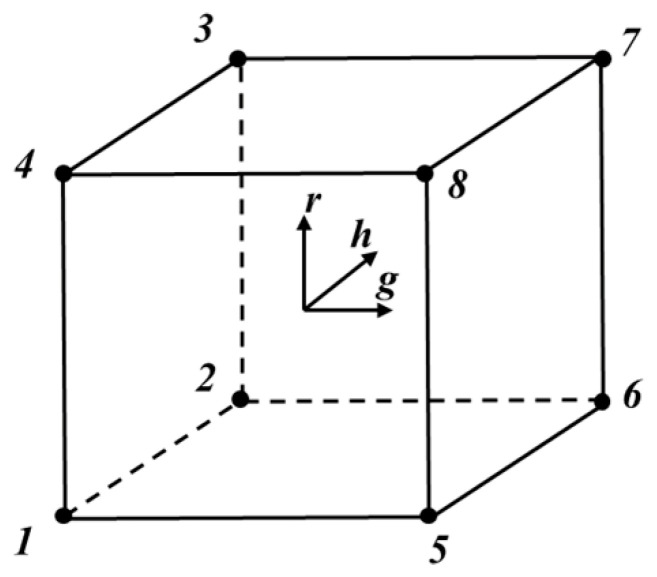
Local node numbering of the first-order brick element in the natural coordinate system.

**Figure A2:**
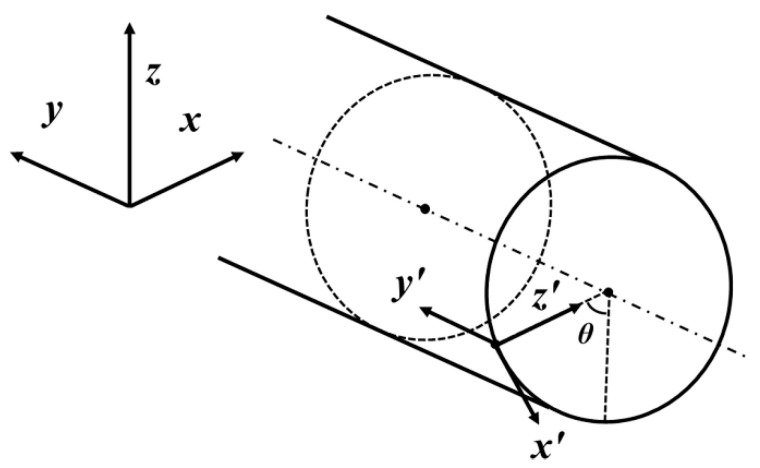
Schematic of local coordinate system for contact calculation.

## Data Availability

The data presented in this study are available on request from the corresponding author.

## Appendix A. Shape Function of the First-Order Brick Element

The first-order brick element with eight nodes (local node numbering is shown in Figure A1) was adopted to model the cold-rolled plate. The shape function of the first-order brick element in the natural coordinate system is given by:

(A1) $$N=\left[\begin{array}{c} \begin{array}{c} N^{\left(1\right)}\\ N^{\left(2\right)}\\ N^{\left(3\right)} \end{array}\\ \begin{array}{c} N^{\left(4\right)}\\ N^{\left(5\right)}\\ N^{\left(6\right)} \end{array}\\ \begin{array}{c} N^{\left(7\right)}\\ N^{\left(8\right)} \end{array} \end{array}\right]=\frac{1}{8}\times \left[\begin{array}{c} \begin{array}{c} \left(1-g\right)\left(1-h\right)\left(1-r\right)\\ \left(1-g\right)\left(1+h\right)\left(1-r\right)\\ \left(1-g\right)\left(1+h\right)\left(1+r\right) \end{array}\\ \begin{array}{c} \left(1-g\right)\left(1-h\right)\left(1+r\right)\\ \left(1+g\right)\left(1-h\right)\left(1-r\right)\\ \left(1+g\right)\left(1+h\right)\left(1-r\right) \end{array}\\ \begin{array}{c} \left(1+g\right)\left(1+h\right)\left(1+r\right)\\ \left(1+g\right)\left(1-h\right)\left(1+r\right) \end{array} \end{array}\right]$$

where $g_{i}$, $h_{i}$, and $r_{i}$ are the coordinates of node *i* in the natural coordinate system.

## Appendix B. Local Coordinate System for Contact Calculation

As shown in Figure A2, when the angle between the $z^{\prime }$ axis of the local coordinate system and the z axis of the global coordinate system is θ, the transformation from the local to the global coordinate system is given by:

(A2) $$\left[\begin{array}{c} x\\ y\\ z \end{array}\right]=\left[\begin{array}{ccc} \cos\theta & 0 & \sin\theta \\ 0 & 1 & 0\\ -\sin\theta & 0 & \cos\theta \end{array}\right]\left[\begin{array}{c} x^{\prime }\\ y^{\prime }\\ z^{\prime } \end{array}\right]$$

The contact behaviors are calculated in the local coordinate system and then converted to the global coordinate system using Equation (A2) when solving the equilibrium equations mentioned in Section 4.
